# Cost per QALY (Quality-Adjusted Life Year) and Lifetime Cost of Prolonged Mechanical Ventilation in Taiwan

**DOI:** 10.1371/journal.pone.0044043

**Published:** 2012-09-06

**Authors:** Mei-Chuan Hung, Hsin-Ming Lu, Likwang Chen, Ming-Shian Lin, Cheng-Ren Chen, Chong-Jen Yu, Jung-Der Wang

**Affiliations:** 1 Department of Public Health, National Cheng Kung University College of Medicine, Tainan, Taiwan; 2 Division of Preventive Medicine and Health Services Research, Institute of Population Health Sciences National Health Research Institutes, Zhunan, Miaoli County, Taiwan; 3 Institute of Public Health, School of Medicine, National Yang-Ming University, Taipei, Taiwan; 4 Department of Internal Medicine, Chia-Yi Christian Hospital, Chiayi, Taiwan; 5 Departments of Internal Medicine, National Taiwan University Hospital, Taipei, Taiwan; 6 Departments of Internal Medicine and Occupational and Environmental Medicine, National Cheng Kung University Hospital, Tainan, Taiwan; Tehran University of Medical Sciences, Iran (Islamic Republic of)

## Abstract

**Introduction:**

Patients who require prolonged mechanical ventilation (PMV) are increasing and producing financial burdens worldwide. This study determines the cost per QALY (quality-adjusted life year), out-of-pocket expenses, and lifetime costs for PMV patients stratified by underlying diseases and cognition levels.

**Methods:**

A nationwide sample of 50,481 patients with continual mechanical ventilation for more than 21 days was collected during 1997–2007. After stratifying the patients according to specific diagnoses, a latent class analysis (LCA) was performed to categorise PMV patients with multiple co-morbidities into several homogeneous groups. The survival functions were estimated for individual groups using the Kaplan-Meier method and extrapolated to 300 months through a semi-parametric method. The survival functions were adjusted using an EQ-5D utility value derived from a convenience sample of 142 PMV patients to estimate quality-adjusted life expectancies (QALE). Another convenience sample of 165 patients was used to estimate the out-of-pocket expenses. The lifetime expenditures paid by the single-payer National Health Insurance (NHI) system and patients' families were estimated by multiplying average monthly expenditures by the survival probabilities and summing the values over lifetime.

**Results:**

PMV therapy costs more than 100,000 U.S. dollars (USD) per QALY for all patients with poor cognition. For patients with partial cognition, PMV therapy costs less than 56,000 USD per QALY for those with liver cirrhosis, intracranial or spinal cord injuries, and 57,000–69,000 USD for patients with multiple co-morbidities under age of 65. The average lifetime cost of PMV was usually below 56,000 USD. The out-of-pocket expenses were often more than one-third of the total cost of treatment.

**Conclusions:**

PMV treatment for patients with poor cognition would cost more than 5 times Taiwan's GDP (gross domestic products), or less cost-effective. The out-of-pocket expenses for PMV provision should also be considered in policy decision.

## Introduction

The number of patients who require prolonged mechanical ventilation (PMV) is rapidly increasing worldwide, which is likely due to an aging population with multiple co-morbidities and to the increasing availability and effectiveness of life-sustaining technologies [1]–[3]. Many patients require continuous respiratory support or even readmission after they have been transferred to a rehabilitation facility, which creates a tremendous strain on healthcare resources and often places a significant financial burden on patients' families [4]. To address these concerns, Taiwan's National Health Insurance (NHI) system drafted a prospective payment program in 1998 that was implemented in 2000 [5] and encouraged integrated care for chronically mechanically ventilated patients, in an effort to decompress crowded intensive care units (ICUs). The program covered mechanical ventilator care in the following step-down sequence: ICUs (acute stage, <21 days, fee-for-service); respiratory care centres (subacute care for ventilator weaning, up to 42 days, per diem); respiratory care wards (chronic stage requiring long-term care, per diem), and homecare services (a stable stage with care provided by family caregivers, per diem). Although this step-down payment schedule immediately relieved crowding in ICUs throughout the country, the total number of PMV patients in Taiwan climbed to 30,000 in 2005 and accounted for 7.5% of the total NHI healthcare expenditures. The long-term provision of mechanical ventilatory support was rapidly becoming one of the major threats to the sustainability of the NHI [5]–[6], making it incumbent upon all the stakeholders to seek out alternative approaches to end-of-life care provision.

Most countries with national health insurance systems have employed various economic analyses to improve overall cost-effectiveness and to control both costs and expenditures [7]–[9]. The cost-effectiveness ratio (CER), which quantifies how many dollars are spent per QALY (quality-adjusted life year) gained, has been widely adopted in the U.K. and by many European countries. To be fair for comparison in economic analysis, we implement the principles of generalized cost-effectiveness analysis, advocated by the World Health Organization (WHO) [10], which estimates the health benefit gained from each treatment technology T compared with no treatment (i.e., natural history). Although this study intends to estimate the CER for PMV in Taiwan, we are concerned that allowing the simple maximisation of cost per QALY to dictate resource allocation would be unfair to people with a short life expectancy and/or poor quality of life [11]. Thus, we set out to quantify the lifetime costs of adopting PMV, which could be fairly compared for different technologies in saving a life and potentially interpreted as that every person is equally entitled to access healthcare services up to his/her life expectancy [12]. In addition, because the information of lifetime out-of-pocket expenses for the PMV therapy is very crucial to the patients and their families, such data were collected to assist clinical decision making.

Based on the results of our previous study of the life expectancy and quality of life in PMV patients [13]–[15], we further integrated the survival function with the healthcare expenditures paid by the NHI and collected out-of pocket expenses in this study to estimate the lifetime cost and the cost per QALY for patients undergoing PMV. The results were stratified by the patients' underlying pathology and cognition levels.

## Subjects and Methods

### Study population and datasets

**Figure 1:**
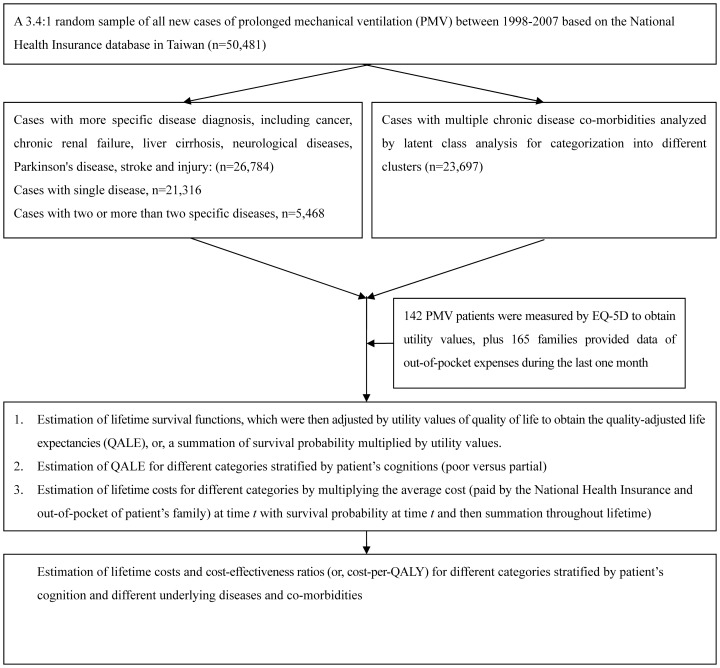
Flow chart of the computation process for lifetime costs and cost-per-QALY (quality-adjusted life year).

The current study was approved by the Institutional Review Board of the National Taiwan University Hospital (IRB number: 200912072R). A nationwide sample of 50,481 patients who were over the age of 17 who had received continual mechanical ventilation for more than 21 days during 1998–2007 was collected [15]. The reimbursement data file was obtained from the NHI and transformed into a research database by the National Health Research Institutes (Chunan, Taiwan) [16]. The identification numbers of all the individuals in the reimbursement data file were encrypted for privacy protection purposes. These files contained detailed demographic information and data on the healthcare utilisation for each patient, including all dates and payments for outpatient visits, hospitalisations, prescriptions, diagnoses, and interventional procedures. The records for each inpatient hospitalisation included up to 5 diagnoses, which were coded according to the International Classification of Diseases (Ninth Revision). After stratifying the patients according to specific diagnoses, a latent class analysis (LCA) was performed to categorise PMV patients with multiple co-morbidities within different age groups into several homogeneous groups, the results of which are summarised in Figure 1. Details of this method can be found in our previous study [15].

### Survival analysis and extrapolation to estimate life expectancy

Each new patient who fulfilled the criteria for PMV was followed starting on day 21 of PMV treatment and continuing until he/she was deceased or censored on 31 December 2007. The lifetime survival of PMV patients (up to 300 months) was obtained via linear extrapolation of a logit-transformed curve of the survival ratio between the study population and an age- and gender-matched reference population. Background population measures were generated by the Monte Carlo method using life tables of the general population of Taiwan.

The full methods and mathematical proof assuming a constant excess hazard, have been described in our previous reports [15], [17]–[21]. To facilitate the computation of the 300-month extrapolation, we used iSQoL, a software program that is based on the *R* statistical package for QALE estimation and can be downloaded for free [22].

### Quality of life data measured via EQ-5D and estimation of quality-adjusted life expectancies (QALE)

To estimate the quality of life (QOL) utility function for PMV patients, we recruited a convenient, cross-sectional sample of 142 PMV subjects [13] during 2008 and 2009 who, after 21 days, continued to receive care from five institutions in Taiwan. Informed consent was obtained from every patient or his/her family caregiver. For subjects who were able to communicate effectively and achieved a score of greater than 15 on the Mini-Mental Status Examination (MMSE) [23]–[24], we assessed QOL by administering the EQ-5D during direct face-to-face interviews. When a patient's level of cognition was too poor for him/her to communicate with the interviewer, family caregivers and nurses were used as proxies [25]–[28]. We have summarised the methods for validating these measurements elsewhere [13]. The duration-to-date for each patient is defined as the time interval between the 21^st^ day after PMV initiation to the time of EQ-5D assessment. A cross-sectional sample of PMV patients was obtained, and a kernel-type smoothing method (using a moving average of the proximal 10%) was performed to estimate the mean QOL throughout the follow-up time period of 9 years [20], [21]. The QOL value after 9 years was assumed to be the same at the end of the follow-up period.

The lifetime survival probabilities along the duration-to-dates were multiplied (or adjusted) with the EQ-5D-measured QOL values to obtain a quality-adjusted survival curve. The total area under this curve was the QALE with QALY as the unit [21].

### Lifetime healthcare expenditures paid by the NHI

We calculated the lifetime healthcare expenditures by the NHI for the 50,481 PMV patients from the 21st day of PMV treatment to the end of life and including the cost of both inpatient and outpatient care. The calculation process was as follows: for each diagnosis-specific group, we summed the average monthly expenditures for each patient and divided the aggregate by the number of PMV patients who were still alive during each month to estimate the average monthly costs to the NHI. Annual NHI expenditures were adjusted to the 2010 Consumer Price Index (CPI). We did not discount after 2010, because PMV patients rarely survive for more than 2–3 years. The total average monthly expenditures were multiplied by the monthly survival probabilities for each diagnosis-specific group over the course of a lifetime, and all values were summed to obtain the lifetime healthcare expenditure within each disease category. In Taiwan, no specific price index has been constructed for calibrating NHI expenditures on health services. Nevertheless, the Taiwanese government occasionally adjusts NHI payment schemes based on the consumer price index and the prices of selected services.

### Lifetime out-of-pocket expenses

This survey was approved by the Institutional Review Board of the Chia-Yi Christian Hospital (IRB number: 098050). To estimate the out-of-pocket money paid by patients and their families, we recruited convenient, cross-sectional 165 PMV subjects during 2009–2010 who received long-term care (>21 days) at eight institutions throughout Taiwan. A written informed consent was obtained from every patient or his/her family caregiver. We asked family caregivers to estimate their relevant expenses over the last month, including the use of supplementary healthcare-related resources not covered by the NHI, non-healthcare related resources, and any loss of salary associated their caregiving responsibilities. The sum of these expenses was calculated by a kernel smoothing method to obtain average monthly expenses along time *t* (duration-to-date) and then multiplied by monthly survival probabilities. The sum of these values represented the average lifetime out-of-pocket expenses for each diagnosis-specific group.

### Estimating cost per QALY for different diagnosis groups

The CER of PMV treatment for each diagnosis group was calculated using the following formula: Estimated total lifetime cost of PMV treatment per patient/Estimated QALE with PMV treatment per patient = cost-per-QALY. It is practically impossible to find a reference group for PMV patients, because these patients require ventilator everyday for at least 6 hours to sustain their lives and such a ventilation support has continued for more than 21 days. In general, such patients would have died within the same day, had they not been on ventilator.

### Uncertainties, sensitivity analysis, and validation of extrapolation method

We used *ex post* approach instead of the conventional *ex ante* approach. Our survival data were real follow-up for 10 years and the healthcare expenditures were retrieved from the reimbursement data files of the NHI plus adjustment for the 2010 CPI. We also calculated the standard errors of the means by the bootstrap method for 100 repeated samples in these parameters, including life expectancy, quality of life, quality adjusted life expectancy (QALE) and lifetime cost. However, uncertainties still exist on the different quality of life and extrapolation method, as we have reclassified the PMV patients according to different underlying diseases and/or co-morbidities. In general, the uncertainty on the utility value of QOL depends heavily on the level of patient's cognition [13], [14]: for instance, approximately 62% of them suffer from cognitive impairments and poor quality of life [13]. Therefore, we directly performed estimation as a sensitivity analysis for QALE and cost per QALY by stratifying PMV patients into partial versus poor cognition. In addition, a validation study was performed on the extrapolation method [15], which is summarized as follows:

We selected subcohorts of patients received PMV between 1998 and 2001, and assumed that these cohorts were only followed until the end of 2001 and then extrapolated these results to the end of 2007. Then, we compared the semiparametric predictions with the Kaplan-Meier (K-M) estimates of the follow-ups from 1998 to 2007. Assuming that the K-M estimates are the gold standard, we calculated the relative biases for subcohorts stratified by different underlying diseases and co-morbidities.

## Results

**Figure 2:**
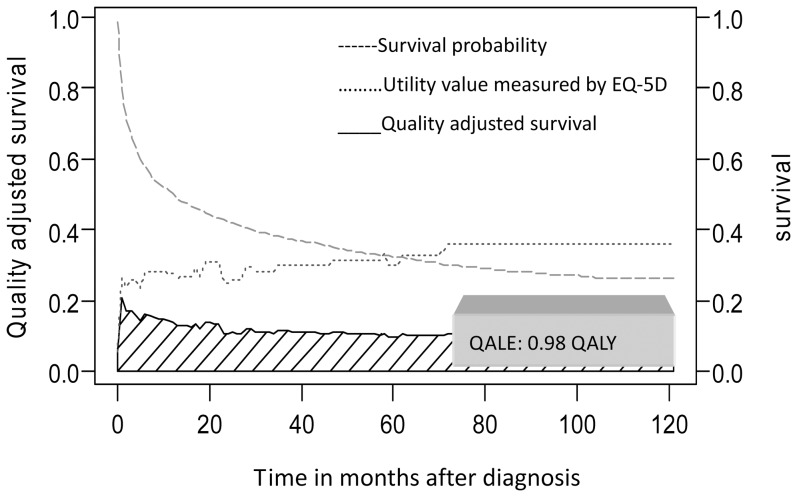
Quality adjusted survival for patients undergoing PMV (prolonged mechanical ventilation) with partial cognition after adjustment for survival function (N = 50,481) with the utility values of quality of life measured with EQ-5D (N = 55). The result of QALE (quality-adjusted life expectancy) of an average patient was 0.98 QALY by summing the areas under the quality-adjusted survival curve.

**Figure 3:**
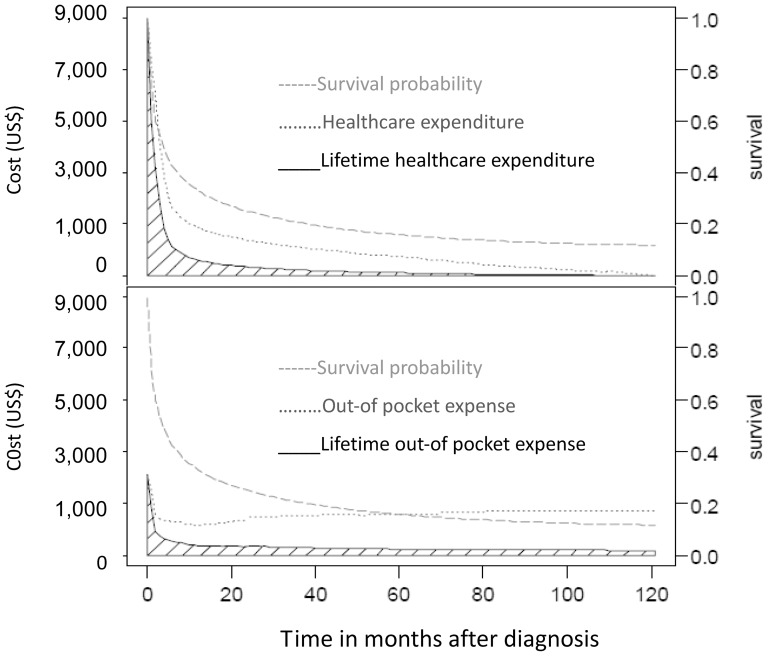
The average lifetime healthcare expenditure paid by the National Health Insurance (NHI) for patients undergoing PMV. (prolonged mechanical ventilation) was calculated by multiplying the monthly average paid by the NHI (N = 50,481) with the corresponding survival probability and then summed up throughout life, as indicated by the shaded areas of the upper panel. The lifetime out-of pocket expense for PMV patients was obtained by multiplying the monthly average out-of pocket expense (estimated by kernel smoothing method on a convenient sample of 165 patients) with the corresponding survival probability and then summed up (shaded areas of the lower panel). The expected lifetime cost of a PMV patient is the total sum of these two shaded areas.

A total of 50,481 new patients receiving PMV were included during the 10-year study period, with a mean age of 72±14.5 years. The demographic and clinical characteristics of the study population were relatively similar to those of the two convenience sample populations that were assessed to measure quality of life and to determine out-of-pocket expenses, as summarised in Table 1. The QALE for patients with partial cognition was 0.98 QALY (Figure 2), while the QALE for patients with poor cognition was 0.42 QALY. The average lifetime cost for a PMV patient was 60,025 USD, of which 32,352 USD was paid by the NHI and 27,673 by the patient and/or family (Figure 3 and Figure S1). In general, out-of pocket expenses paid by the patient and/or patient's family comprised more than one-third of the total cost of care, although some diagnosis-related variations did exist (Tables 2 and 3).

**Table 1:** Characteristics of patients undergoing prolonged mechanical ventilation (PMV) and 2 cross-sectional samples for assessing quality of life (QOL) and out-of-pocket expenses.

Characteristics	Cohort of PMV	Cross-sectional sample of QOL	Cross-sectional sample of out-of-pocket expenses
Calendar years of collection	1998–2007	2008–2009	2009–2010
Total No. of patients	50,481	142	165
Mean age in years (SD)*	72 (15)	75 (12)	76 (12)
<64 yrs	11,670 (23%)	20 (14%)	19 (12%)
65–74 yrs	12,203 (24%)	23 (16%)	28 (17%)
75–84 yrs	18,331 (36%)	53 (38%)	66 (39%)
>85 yrs	8,277 (17%)	46 (32%)	52 (32%)
No. (%) of females	20,193 (40%)	61 (43%)	90 (54%)
No. (%) of hypertension	6,563 (13%)	40(28%)	-
No. (%) of diabetes mellitus	10,096 (20%)	36(25%)	-
No. (%) of CVA†	10,097 (20%)	26(18%)	-
No. (%) of COPD‡	7,573(15%)	46(32%)	-
No. (%) of asthma	1,515 (3%)	6(4%)	-
No. (%) of liver cirrhosis	505 (1%)	3(2%)	-

* SD: standard deviation.

† CVA: cerebral vascular accident.

‡ COPD: chronic obstructive pulmonary disease.

**Table 2:** Lifetime cost and cost per QALY (quality-adjusted life year) for patients undergoing prolonged mechanical ventilation in Taiwan, stratified by underlying diseases (Dis.), with sensitivity analysis of quality-adjusted life expectancy (QALE) under different states of cognition.

	No. of cases	Life expectancy (yrs)(SE)*	QALE (QALY)(SE)*		Lifetime cost ($US)		Cost per QALY	
			partial cognition	poor cognition	healthcare expenditure	out of pocket	partial cognition	poor cognition
Overall	21,316	3.26 (0.10)	0.93 (0.17)	0.47 (0.09)	32,873	30,450	68,089	134,730
Cancer	5,367	1.49 (0.08)	0.46 (0.08)	0.20 (0.03)	15,835	13,931	64,708	148,829
Renal failure	2,032	1.32 (0.12)	0.40 (0.09)	0.18 (0.04)	24,253	12,237	91,224	202,720
Liver cirrhosis	1,478	3.50 (0.37)	1.15 (0.22)	0.50 (0.13)	19,652	32,568	45,409	104,440
Degenerative nervous Dis.†	378	4.08 (0.60)	1.28 (0.25)	0.56 (0.14)	78,622	36,898	90,250	206,286
Parkinson's Dis.†	341	2.01 (0.27)	0.59 (0.14)	0.26 (0.07)	44,708	17,461	105,371	239,110
Stroke	6,765	3.32 (0.13)	1.05 (0.20)	0.46 (0.09)	42,452	29,932	68,938	157,358
<64 yrs	1,955	5.24 (0.30)	1.60 (0.39)	0.72 (0.13)	54,686	47,487	63,858	141,907
65–74 yrs	1,818	2.93 (0.18)	1.14 (0.16)	0.40 (0.09)	43,350	26,120	60,939	173,676
75–84 yrs	2,176	2.03 (0.11)	0.61 (0.08)	0.27 (0.04)	40,099	17,978	95,209	215,101
>85 yrs	816	1.42 (0.09)	0.49 (0.06)	0.21 (0.03)	30,056	12,486	86,820	202,581
Intracranial or spinal injury	4,955	6.19 (0.17)	2.04 (0.39)	0.89 (0.18)	43,090	56,806	48,969	112,242
<64 yrs	1,949	10.06 (0.51)	3.40 (0.68)	1.47 (0.31)	56,872	92,951	44,065	101,920
65–74 yrs	1,116	3.71 (0.35)	1.18 (0.18)	0.51 (0.09)	38,268	33,415	60,748	140,555
75–84 yrs	1,366	2.64 (0.12)	0.82 (0.12)	0.33 (0.07)	33,405	23,720	69,665	173,106
>85 yrs	524	1.51 (0.09)	0.52 (0.09)	0.22 (0.03)	30,213	13,319	83,715	197,873
Cases with >2 specific Dis.†	4,772	2.90 (0.13)	0.93 (0.17)	0.40 (0.07)	32,137	26,450	62,997	146,467
Cancer and renal failure	165	1.14 (0.33)	0.36 (0.15)	0.16 (0.06)	23,236	10,778	94,482	212,585
Cancer and others	1,609	1.82 (0.24)	0.58 (0.13)	0.26 (0.04)	19,773	16,849	63,141	140,853
Renal failure and others	743	1.65 (0.29)	0.52 (0.14)	0.23 (0.07)	26,698	15,484	81,119	183,400

* SE: standard error of the mean.

† Dis. : diseases.

**Table 3:** Lifetime cost and cost per QALY (quality-adjusted life year) for patients undergoing prolonged mechanical ventilation in Taiwan, stratified by different co-morbidities and age, with sensitivity analysis of quality-adjusted life expectancy (QALE) under different states of cognition.

	Number of cases	Life expectancy (yrs) (SE)*	QALE (QALY) (SE)*		Lifetime cost ($US)		Cost per QALY	
			partial cognition	poor cognition	healthcare expenditure	out of pocket	partial cognition	poor cognition
<65 yrs								
Heart diseases	616	4.97 (0.63)	1.61 (0.41)	0.70 (0.19)	47,230	45,463	57,574	132,419
Septicemia/Shock	919	4.42 (0.59)	1.22 (0.23)	0.64 (0.14)	27,797	40,663	56,115	106,969
Urinary tract infections/Shock	197	4.77 (0.98)	1.43 (0.35)	0.62 (0.18)	54,799	43,487	68,731	158,525
COPD†	1,788	5.18 (0.28)	1.66 (0.24)	0.72 (0.14)	59,284	46,875	63,951	147,444
65–74 yrs								
Heart diseases	1,074	2.49 (0.24)	0.77 (0.14)	0.34 (0.07)	30,948	22,496	69,408	157,189
Septicemia/Shock	1,824	2.08 (0.16)	0.65 (0.09)	0.28 (0.04)	24,846	18,825	67,187	155,969
COPD†	2,499	2.49 (0.15)	0.76 (0.11)	0.33 (0.06)	36,931	22,214	77,822	179,227
75–84 yrs								
Heart diseases	1,404	1.78 (0.17)	0.54 (0.11)	0.24 (0.05)	25,881	16,049	77,648	174,707
Septicemia/Shock	2,856	1.60 (0.10)	0.49 (0.03)	0.22 (0.05)	22,427	14,590	75,544	168,258
COPD†	4,142	2.05 (0.10)	0.63 (0.09)	0.28 (0.05)	32,472	18,496	80,902	182,029
Respiratory diseases	1,345	2.13 (0.13)	0.66 (0.13)	0.29 (0.05)	31,907	19,202	77,438	176,238
>85 yrs								
Heart diseases	870	1.43 (0.12)	0.41 (0.05)	0.18 (0.03)	25,015	12,660	91,891	209,306
Septicemia/Shock	1,359	1.07 (0.09)	0.31 (0.05)	0.14 (0.02)	19,194	11,436	98,808	218,789
COPD†	2,804	1.46 (0.06)	0.42 (0.06)	0.19 (0.03)	26,736	12,895	94,361	208,587

* SE: standard error of the mean.

† COPD: chronic obstructive pulmonary disease.

**Figure 4:**
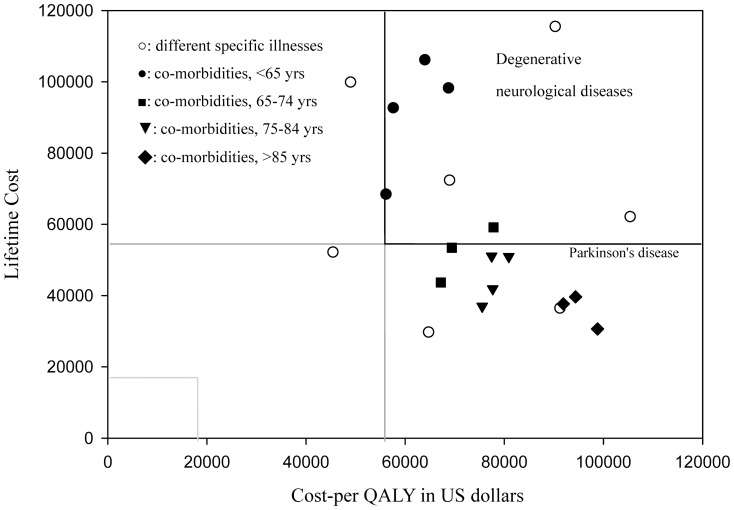
Plot of association between the cost-per-QALY (quality-adjusted life year) gained and the lifetime cost for different diagnosis groups of PMV patients with partial cognition. , stratified by different specific illnesses [○] and multiple co-morbidities (indicated by sub-age groups, <65[•], 65–74[▪], 75–84[▾], and >84[□] years old). The GDP (gross domestic product) of Taiwan in 2010 was 18,588 US dollars, below which the cost-per-QALY was considered as cost-effective; between 1–3 times the GDP per QALY gained was considered moderate cost-effective, and above 3 times the GDP per QALY gained was not cost-effective. The figure indicates there are many conditions showing a lifetime cost less than 3 times the GDP, although their costs-per-QALY were above that figure.

After stratification by different underlying causes, we found that most lifetime costs for PMV were below 56,000 USD except for patients who survived for more than 3–4 years, which often included patients with degenerative neurological diseases, stroke, intracranial or spinal cord injuries or multiple co-morbidities who were younger than 65 years old (Tables 2 and 3). The results of the sensitivity analysis also revealed that PMV therapy costs over 100,000 USD per QALY for patients with poor cognition. Figure 4 depicts the association of cost per QALY and lifetime cost for PMV patients with partial cognition under different disease categories, illustrating that the lifetime cost associated with treating most PMV patients is less than 3 times Taiwan's GDP.

### QALE, lifetime costs, and cost per QALY for PMV patients with specific underlying diseases

The QALE of PMV patients with partial cognition was generally 2 times greater than that of patients with poor cognition. The QALE of patients with injuries or liver cirrhosis was generally better than that of patients with chronic renal failure or cancer. When a patient suffered from both cancer and chronic renal failure, the QALE was the lowest, with a QALY of 0.16 and 0.36 for patients with poor and partial cognition, respectively (Table 2). For patients with partial cognition, PMV therapy costs less than 56,000 USD per QALY in patients with liver cirrhosis or intracranial or spinal cord injuries; it costs approximately 57,000–69,000 USD per QALY for patients with cancer or stroke.

### QALE, lifetime costs, and cost per QALY for PMV patients with multiple co-morbidities

Of the 23,697 PMV patients with multiple co-morbidities, the latent class model typically yielded 3–4 clusters within each age stratum, including heart disease, septicaemia/shock, chronic obstructive pulmonary disease (COPD) and/or others (e.g., urinary tract infections) (Table 3). The QALE of PMV patients with COPD was generally better than those of other clustered groups, especially in patients with septicaemia/shock (Table 3). Only patients who were younger than 65 years old with partial cognition cost less than 68,000 USD per QALY gained.

### Validation of the semi-parametric method for long term extrapolation of survival function

The results obtained to validate our semi-parametric method show that the relative biases of extrapolation from the end of the 4^th^ year to that of the 10^th^ year were all below 20%. Among them, the relative biases of most PMV patients with a specific diagnosis ranged between 0.9% and 5.5%, or, are more accurate as they were generally more homogeneous (Table S1).

## Discussion

### Summary of main findings

To our knowledge, this study is the first to analyse a national PMV dataset and estimate the cost per QALY stratified by cognition level, age, and different clusters of diagnoses. Most importantly, our estimates took into account both healthcare expenditures reimbursed by the Taiwanese NHI and out-of pocket expenses paid by patients' families, which is critical information for any who have a stake in the care provision and decision-making processes surrounding the use of PMV. In other words, while the varied costs per QALY for patients with different cognitive states across different diagnosis-specific groups may guide NHI managers in their decision-making process regarding resource allocation, they also alert the PMV patient and his/her family to the extent of the financial burden that is likely to accompany any decision to keep their loved one chronically mechanically ventilated. Because all co-payments are waived for PMV patients in Taiwan and the healthcare expenditures paid by the NHI from 1998–2007 were fully adjusted to the 2010 CPI, our estimation of lifetime expenditures for the NHI should be accurate. Moreover, our validation study showed that the relative biases of extrapolated estimates were usually below 5–20%. As these patients rarely survive for more than 2–3 years (Tables 2 and 3), our estimation of lifetime survival function would be generally precise. To control for any potential confounding between the underlying disease and level of cognition, we stratified all patients by their specific illnesses (Table 2), the presence of co-morbidities and age (Table 3) and then further by cognition level. As the quality of life values and out-of-pocket expenses were determined from relatively representative convenience samples (Table 1), we tentatively conclude that our CER analysis of PMV provides the best available information on care expenditures and would be useful to all relevant stakeholders in their deliberations about resource allocation in Taiwan.

### Interpretation with reference to other research

As previously reviewed by Dr. Cooke [4], there have been several studies quantifying cost per QALY for PMV. The estimation based on Markov model was approximately $103,100 USD per QALY in 2011 and varied tremendously with age and mortality risk [4], [29]. For a fair comparison, we adopted the criterion suggested by WHO-CHOICE (CHOosing Interventions that are Cost Effective) and used 1 to 3 times the GDP as the threshold for cost-effectiveness [30]. Because Taiwan's GDP per capita in 2010 was 18,588 USD [31], providing PMV to all patients with poor cognition would cost more than 5 times the GDP. For patients who were able to communicate or retained partial cognitive function, we found that PMV therapy for those who suffered from cancer or from multiple co-morbidities who were also less than 65 years of age cost 3 to 3.7 times the GDP per QALY. In contrast, PMV therapy generally costs less than 56,000 USD (less than 3 times the GDP) per QALY for patients with liver cirrhosis, intracranial or spinal cord injuries.

If we consider that every person is entitled to receive lifesaving treatment, such as PMV, up to one's life expectancy, then we should consider the estimated lifetime cost of care provision (Tables 2 and 3), which is generally below 56,000 USD, except for patients with degenerative neurological diseases, stroke, intracranial or spinal cord injuries or patients with multiple co-morbidities who are younger than 65. In other words, the care expenditures for most PMV patients amount to less than 3 times the GDP for one life, as depicted on the right lower quadrant of Figure 4 for PMV patients with partial cognition across different strata. Are we not willing to sustain the life of a PMV patient with partial cognition for a small amount of time, if that is, indeed, the patient's wish? However, if we accept Professor Daniels' position that every citizen is entitled to access healthcare services up to his/her life expectancy [12], should not we then consider a possible higher priority for a health condition that incurs a lifetime cost smaller than the presumably established threshold cost per QALY?

From both a medical and cost-effective point of view, there is value in investing in resources that more effectively wean patients from ventilator dependency. At the same time, it is important to avoid spending too much money in ways that simply prolong the dying process without improving patients' quality of life. Hence, it might be useful for the NHI to establish a ceiling value for lifetime costs under the budget constraint. There will be an unavoidable trade-off between cost-effectiveness and fairness in distribution under any system of reimbursement for PMV. Therefore, we recommend that such a decision be made during the deliberation process and involve all stakeholders [32]. Moreover, the cost per QALY and lifetime cost must be determined for health conditions other than those requiring PMV to provide a more extensive awareness of the cost-effectiveness of standard healthcare practices. For example, would we be willing to spend more on child health, vaccinations, and preventive care, among other services? Such a decision will involve a difficult balancing act between assessing what is good for patients and their families, offering effective incentives to the providers of healthcare services, ensuring the sustainability of the NHI, and considering what is good for society at large. We hope that our findings may serve as a call to the public to contemplate and openly discuss the principles of health resources distribution under a national health insurance system, including the provision of PMV care.

### Potential limitations

Our study has some limitations. First, because there are no MMSE data in the NHI database, we were unable to directly quantify the QALE for each individual condition. The estimation in this study is, in fact, a sensitivity analysis for the two largely distinct mental health states characterised by the patient's cognitive condition. Because the utility value for patients with poor consciousness is generally low [13], [33] and the usual life expectancy for PMV patients is less than 2–3 years [15], [29], the potential bias associated with using this estimation should be minimal. Second, during the lifetime extrapolation of the QOL function, it was assumed that the patients remained at the same QOL as what was measured at the end of the follow-up period. Such an assumption could result in an overestimation of the QALE because the actual QOL might gradually decline with age and increasing co-morbidity [34]–[35]. However, because the QOL of the PMV patients was very poor even when they retained partial cognitive function, our estimates probably only slightly overestimate lifetime QOL, at most, and likely would not alter policy decision making. While the QOL values might be relatively uniform within Taiwan, they are profoundly influenced by a variety of factors (e.g., culture, age, economic status, family support environment, religion, etc.) and are otherwise likely to be very different from the measurements in a more diverse society. Thus, they may not be appropriately extrapolated across different societies and/or cultures, and precautions must be taken before any attempt is made to adopt our findings for application in another country.

## Conclusions

The cost per QALY for PMV varies greatly depending on the cognition level of patients and the underlying disease and co-morbidities. Maintenance treatments for PMV patients with poor cognition usually cost more than 5 times the GDP of Taiwan, or less cost-effective. Because the lifetime cost seems to be more equitable for those with a short life expectancy and/or poor quality of life than the cost per QALY, we propose that it also be considered in future deliberations about healthcare resources allocation. Moreover, since out-of-pocket expenses seem to occupy more than one-third of the total costs, patients and their families must be informed of financial burden during the clinical decision-making process.

### What is already known on this topic?

The cost per QALY of prolonged mechanical ventilation varies according to the patient's age, underlying causes of respiratory failure and risk of mortality. In general, PMV for very elderly and very sick patients is a low-value way to use finite resources.

### What this study adds

1. The cost per QALY for PMV treatment is usually over 100,000 US dollars or 5 times the GDP of Taiwan for all patients with poor cognition.
2. Out-of-pocket expenses are usually more than one-third of the total PMV-related costs in Taiwan, although the exact figures may vary within different cultures and societies.
3. Lifetime costs for a specific illness might be lower than the threshold cost per QALY presumably established in a country for different conditions and both could be considered together in future deliberations to balance equity and efficiency in healthcare resource allocation.

## Supporting Information

Figure S1 **Monthly average paid by the NHI (N = 50,481) after implementation of PMV are summarized in the upper panel, while that of out-of pocket expenses (estimated by kernel smoothing method on a convenient sample of 165 patients) are summarized in the lower panel with the original surveyed data and depicted.**
(TIFF)

Table S1 **Validation of extrapolation method by comparing the Kaplan-Meier (K–M) estimates of 10-year follow-up of PMV patients and those based on the survival of first 4-year and extrapolated to 10-year.**
(DOCX)
