# Supplementary material for: Cost per QALY (Quality-Adjusted Life Year) and Lifetime Cost of Prolonged Mechanical Ventilation in Taiwan
Source: PLoS One. 2012 Sep 6;7(9):e44043. doi: 10.1371/journal.pone.0044043 (PMC3435371; doi:10.1371/journal.pone.0044043)
Supplement: Table S1 — Validation of extrapolation method by comparing the Kaplan-Meier (K–M) estimates of 10-year follow-up of PMV patients and those based on the survival of first 4-year and extrapolated to 10-year. (DOCX) [file pone.0044043.s002.docx]

**Table S1**

| Age | Diagnosis | Cohort size | Censor rate (%) | [A]10-yr survival based on K-M estimate (SE) * | [B]Extrapolation to 10-yr based on the 4-yr follow up(SE) * | Relative bias (%)^†^ |
| --- | --- | --- | --- | --- | --- | --- |
|  | Cancer | 1,997 | 18.6 | 1.13 (0.06) | 1.14 (0.08) | 0.9 |
|  | Renal failure | 842 | 17.3 | 1.05 (0.09) | 1.07 (0.15) | 1.9 |
|  | Liver cirrhosis | 671 | 27.4 | 2.00 (0.11) | 2.17 (0.17) | 8.5 |
|  | Degenerative nervous disease | 573 | 44.1 | 2.78 (0.13) | 2.83 (0.33) | 1.8 |
|  | Stroke | 2,312 | 36.5 | 2.26 (0.06) | 2.01 (0.11) | -11.1 |
|  | Intracranial or spinal injury | 2,027 | 42.9 | 3.30 (0.08) | 3.12 (0.14) | -5.5 |
| <64 yrs | Heart diseases | 650 | 46.3 | 3.43 (0.28) | 3.27 (0.33) | -4.7 |
|  | COPD^‡^ /Others | 277 | 35.0 | 2.50 (0.26) | 2.69 (0.33) | 7.6 |
| 65-74 yrs | Heart diseases | 915 | 37.3 | 2.10 (0.10) | 2.49 (0.18) | 18.6 |
|  | SP^§^/Shock | 543 | 25.6 | 1.46 (0.21) | 1.35 (0.19) | -7.5 |
|  | COPD ^‡^ /Others | 366 | 30.6 | 2.10 (0.17) | 2.13 (0.26) | 1.4 |
| 75-84 yrs | Heart diseases | 1242 | 32.1 | 1.84 (0.09) | 2.01 (0.17) | 9.2 |
|  | SP^§^/Shock | 386 | 28.8 | 1.77 (0.16) | 1.74 (0.27) | -1.7 |
|  | COPD ^‡^ /Others | 649 | 21.9 | 1.26 (0.09) | 1.16 (0.20) | -7.9 |
|  | Respiratory diseases | 389 | 27.5 | 1.67 (0.11) | 1.47 (0.27) | -12.0 |
| > 85 yrs | SP^§^/Shock | 304 | 21.1 | 1.16 (0.15) | 1.25 (0.17) | 7.8 |
|  | COPD ^‡^ /Others | 692 | 30.4 | 1.79 (0.12) | 1.64 (0.19) | -8.4 |

* SE: standard error of the mean

†: relative bias (%) = ((B-A)/A)*100

‡ COPD: chronic obstructive pulmonary disease

§ SP: septicemia
